# Are Simple Renal Cysts in Childhood Associated With Kidney Stones?

**DOI:** 10.5812/numonthly.2682

**Published:** 2012-09-24

**Authors:** Victor Garcia-Nieto, Francisco Negrete-Pedraza, Marta Lopez-Garcia, Maria Isabel Luis-Yanes

**Affiliations:** 1 Pediatric Nephrology Unit, Hospital Nuestra Señora de Candelaria, Santa Cruz de Tenerife, Spain; 2 Pediatric Nephrology Department, La Raza National Medical Center IMSS, Mexico; 3 Pediatrics Department, Hospital Universitario de Canarias, Tenerife, Spain

**Keywords:** Kidney Calculi, Hypercalciuria

The renal tubules, in their various segments, are mainly responsible for the final composition of urine from glomerular ultrafiltrate. In a complex process involving resorption and secretion mechanisms, the tubules are responsible for retaining substances in the body, some substances are recovered almost 100% such as water and sodium chloride. Tubulopathies are a group of diseases where these selection mechanisms are impaired; they can be simple or complex depending on the number of the dysfunctional mechanisms. Nevertheless, tubules may present both morphological and structural abnormalities. Some of these abnormalities can be congenital (development abnormalities) usually on a genetic basis. Some are malformations (renal multicystic dysplasia), some others hereditary diseases (AR polycystic kidney). Other morphologic tubular diseases can develop after birth as part of genetic diseases such as AD polycystic kidney disease, tuberous sclerosis or medullary cystic disease.

Sometimes renal cysts are unique and, generally, do not compromise the future of the patient. These are known as a simple renal cyst, a disease that is not known whether it is a “benign” malformation itself or an acquired abnormality of uncertain aetiology. Since renal cysts are rarely seen at birth one might assume them to be acquired (1). In the past, they were an occasional finding in the excretory urography requested in cases of abdominal mass or because of the presence of symptoms such as flank pain, hematuria, hypertension or any other urological problem. Currently this diagnosis especially in childhood is made by ultrasound and, in most cases, it is a fortuitous finding. This technique shows a round mass of sharp edges and smooth walls with absence of internal echoes and with ultrasound reinforcement on the posterior wall. They are more common in males, in the upper pole of the left kidney (2).

Although their existence has been known since ancient time (3, 4), so far, there is no theory or hypothesis published based on their aetiology and significance. However, it has been suggested that these cysts would be originated from diverticulas of the distal convoluted tubule or the collector duct (1). In 1930, Hepler suggested that they could be caused by a tubular obstruction which would produce an increase in the size of the pre-existing cyst with age (4).

We noticed that some patients with simple renal cysts were diagnosed with idiopathic hypercalciuria, we initially hypothesized that tubular obstruction would be the result of the presence of crystalline material or microstones inside the collecting duct located in the renal pyramid. Therefore, we conducted an ambispective study in children with simple renal cysts diagnosed by renal ultrasound. We found that these children had the most common metabolic disturbances in urolithiasis; hypercalciuria and hipocitraturia (5). We also look for the frequency of urolithiasis in relatives of first and second grade. We studied 22 patients (12 males, 10 females) from the outpatient clinics of our hospital. The mean age was 5.67 ± 3.03 years (range: 1-13 years). The patients were followed up during 1.77 ± 3.68 years (range: 0-17 years). Surprisingly, 14 of the 22 children (63.6%) had hypercalciuria (n = 9), hypocitraturia (n = 3) or both (n = 2). The frequency of childhood hypocitraturia in healthy controls has not been established but that for hypercalciuria is known to be 2.9-8.6%, much lesser than the 50% we found in our patients (5). There were also familial kidney stones history in 13 children (58.1%), eight with metabolic abnormalities and five without them. The familial frequency for urolithiasis results little more than twice of the observed in our control population (28.1%) (6). In summary, 86.3% of the patients had hypercalciuria, hypocitratura and/or familial history of urolithiasis. We hypothesize that there is a relationship between urolithiasis and simple renal cysts. And our hypothesis is also supported by the results obtained from the study published by Chang et al. in 2007 (7). In their article the frequency of kidney stones in the group with renal cysts (n = 62) was 24.2% versus 11.5% in the control group (n = 515) (P < 0.001). Nevertheless, the authors do not refer any reason to explain the relationship on their results. They admitted that “kidney stones could be a risk factor in the presence of simple renal cysts” (7).

Until recently, when a patient was considered as candidate to develop kidney stones, his genetic predisposition was known once he suffered his first renal colic, usually in adulthood. Except for the very inadequate diets, as in the case of maintained high salt ingestion (8) and/or proteins (9), most of the causes of kidney stones are taken as inherited and therefore from genetic aetiology. This is obvious in the case of oxalosis or cystinuria but also true in the case of idiopathic hypercalciuria (10, 11). Well then, since less than 30 years ago, it has been known that children with idiopathic hypercalciuria, i.e. those who have a bigger risk of kidney stones formation in adulthood, may debut in infancy with signs and symptoms other than those of urolithiasis itself, such as macroscopic or microscopic hematuria (12, 13), sterile leukocyturia (14), dysuria (14, 15, 16), and miccional urgency (14, 16), chronic abdominal pain (17), urinary tract infection (18, 19), blurred urine (17) or nocturnal enuresis (14, 18, 20, 21). This issue of genetic predisposition to form kidney stones has been called pre-lithiasis, but is also true that not all the subjects become to form stones in adulthood, especially if they are careful through appropriate changes in their diet. In summary, our hypothesis is that both entities, simple renal cysts and genetic predisposition to kidney stones (pre-lithiasis) can be related. Simple renal cysts would join to other symptoms and signs that suggest a genetic predisposition to form kidney stones somewhere in a lifetime (6).

Nevertheless, the matter could be even more complex. A similar association between simple renal cysts and urolithiasis have been previously observed in children who have two renal malformations, vesicoureteral reflux (VUR) and ureteropelvic junction obstruction (UPJO). For long time, an association between urolithiasis and congenital anatomical deformities of the genitourinary tract has been described in adults and children (22, 23, 24). The frequency of underlying genitourinary anomalies in children with urolithiasis has been estimated to be 19.1% to 29.8%. The precise pathogenic relationships between urolithiasis and congenital abnormalities of the kidney and urinary tract remain unclear. The pathophysiologic mechanism for the formation and growth of the stones has been related to urine stasis and infection, which is more evident in UPJO. The incidence of urolithiasis in patients with UPJO is 16-44.7% (25), with a 70-fold increased risk for developing kidney stones (26). In a previous survey, Husmann et al. (27) reported that 76% of patients with UPJO and simultaneous non-struvite renal calculi presented an identifiable metabolic abnormality. Hypercalciuria has been the most frequently reported metabolic disturbance in patients with UPJO (26, 27) although others, such as hypocitraturia and hyperoxaluria, have also been found (25, 28). No single of these works mention the origin of hypercalciuria and the other metabolic disturbances causing calculi present in patients with UPJO. We conducted a study that was carried out to find out if children with UPJO have higher prevalence of hypercalciuria and whether their family members were affected by hypercalciuria and/or urolithiasis (29). Hypercalciuria was found in 17/27 children (63%), 15 of them (88%) had a familial history of urolithiasis. Concerning the 10 children without hypercalciuria, seven of them (70%) had a familial history of urolithiasis. The prevalences of both, urolithiasis and hypercalciuria, were not influenced by gender. In summary in concordance with previous data (26, 27), our results showed that the prevalence of hypercalciuria is greater in pediatric patients with UPJO than in the general population. Likewise, the prevalence of urolithiasis in the families of these children is also higher than that in the general population. Hypercalciuria was inherited, apparently as an autosomal dominant trait (29). On the other hand, in a previous report we observed that the prevalence of hypercalciuria was greater in children with VUR than in the general population, and furthermore our findings suggested that hypercalciuria in these patients may have itself genetic origin (30). Recently, it has been confirmed our initial finding about the association between VUR and hypercalciuria (31). The consequence of our two studies (29, 30) has shown that the genetic link between urolithiasis and these congenital abnormalities of the kidney and urinary tract, VUR and UPJO, could be explained by similar genetic mechanisms. Furthermore, our data support the idea that adults with hypercalciuria and urolithiasis, symptomatic or not, are at a higher risk of conceiving children affected by UPJO or VUR and hypercalciuria.

But let’s back to children with renal cysts and their relationship to pre-lithiasis described above (5). Are not the results very similar to what we have described previously in patients with VUR (30) or UPJO (29)? Therefore, our current hypothesis is that simple renal cysts are a tubular development anomaly rather than an acquired secondary lesion.

We know that the matter described in this Editorial can be controversial. For many years in the medical literature one could read that the pathophysiologic mechanism of stone formation in children and adults with congenital urinary tract abnormalities were the urine stasis and infection. We believe that this relationship is far deeper where there must be involved, at least, genetic and metabolic disturbances causing the formation of kidney stones.
